# Attending work or not when sick – what makes the decision? A qualitative study among car mechanics

**DOI:** 10.1186/1471-2458-12-813

**Published:** 2012-09-21

**Authors:** Tone Morken, Inger Haukenes, Liv Heide Magnussen

**Affiliations:** 1 Uni Health, Uni Research, Bergen, Norway; 2 Department of Public Health and Primary Health Care, University of Bergen, Kalfarveien 31, Bergen, N-5018, Norway; 3 Faculty of Health and Social Sciences, Bergen University College, Bergen, Norway

**Keywords:** Sick leave, Work environment, Decision-making

## Abstract

**Background:**

High prevalence of sickness absence in countries with generous welfare schemes has generated debates on mechanisms that may influence workers’ decisions about calling in sick for work. Little is known about the themes at stake during the decision-making process for reaching the choice of absence or attendance when feeling ill. The aim of the study was to examine decisions of absence versus attendance among car mechanics when feeling ill.

**Methods:**

Interviews with 263 male car mechanics from 19 companies were used for the study, analysed by systematic text condensation and presented as descriptions and quotations of experiences and opinions.

**Results:**

Three major themes were at stake during the decision-making process: 1) Experienced degree of illness, focusing on the present health condition and indicators of whether you are fit for work or not; 2) daily life habits, where attending work was a daily routine, often learned from childhood; 3) the importance of the job, with focus on the importance of work, colleagues, customers and work environment.

**Conclusions:**

The car mechanics expressed a strong will to attend work in spite of illness. Knowledge about attitudes and dilemmas in reaching the decision regarding sickness absence or sickness attendance is useful in the prevention of sickness absence.

## Background

High prevalence of sickness absence in countries with generous welfare schemes has generated debates on mechanisms that influence workers’ decisions about calling in sick for work. Several social, psychological and physical factors are associated with sickness absence and attendance [1-8], however, literature regarding the decision process is scarce. To call in sick for work seems to be a decision that workers take within a certain cultural and social framework [9,10]. One study found that problems in workplace relationships or stressful work situations influenced the choice of calling in sick for work [11]. An important decisive factor for sickness absence among offshore catering workers was the individual’s assessment of the severity of the illness [8]. Another study found that the culture of ‘working through illness’ was more common among hospital doctors compared with general physicians and management consultants [12]. Further, Aronsson et al. [13] found the highest presenteeism among female-dominated occupations within health care and education, leaving male-dominated occupations such as welders, mechanics and machine operators with considerable lower rates. Consequently, the value of generalising across occupations may be limited [10,14]. To overcome this barrier there is a need for studies that examine absence and attendance decisions within specific groups.

Car repair shops are dominated by men, mostly young, and the working object (car) as such sets limits for the adjustment of work to health status [15]. A car is a massive object, and the repair process is performed within, under and around the car and can be characterised by the physical closeness to the working object. Also, the working object is not a flexible object and thereby demands bodily flexibility of the worker to make the repair process possible. The car repair industry has developed from being a traditional craft industry to using pneumatic tools, vehicle lifts and computerized diagnostics for engine defects. Despite these improvements, the work is physically demanding, and may be associated with sickness absence [16]. Additionally, in modern car repair shops the repair process is highly accelerated. A car mechanic who solves a problem faster than scheduled gets a bonus in addition to his fixed salary. Consequently, adjustments of work to health by for example working at a slower pace may be difficult to carry out without risk of losing profits. A study among car mechanics found a higher risk of being absent from work when reporting shoulder and back problems [17]. Certain levels of musculoskeletal pain are inconsistent with performing vehicle repair work and may influence the decision of sickness absence or continued attendance. Probably the specific work environment of car mechanics influences the threshold for absence or attendance, especially when health status interferes with work performance.

In Norway, workers can apply self-certified sickness absence (12–24 days per year) or physician-certified sickness absence, dependent on the length of the absence. Employers are obliged to cover 100% of regular salary during the first 16 days of sickness absence, before the National Insurance Administration takes over the payment (100% covered) for a total of 12 months. Due to the generous benefit system, non-monetary work incentives may be important for workers’ decisions of absence or attendance. However, we have little knowledge of how these decisions are reached by the individual [18] and whether the nature of work in specific occupations influence these decision processes.

The aim of this study was to examine decisions of absence versus attendance among car mechanics when feeling ill. The study may add insight into areas of consideration in the process of choosing absence or attendance.

## Methods

We obtained individual statements of decisions regarding absence or attendance when feeling ill from 263 male car mechanics in 19 companies through semi-structured face-to-face interview. The Regional Committee for Medical Research Ethics in Western Norway approved the study.

The study was performed in 2007 as part of a larger semi-structured interview study of sickness absence among employees in car repair shops [19]. Invitations were sent to all car repair shops with membership in the Occupational Health Service owned by the Automobile Association in Bergen, Norway (n = 20). One company had an unexpected visit from a certification bureau on the scheduled day for interview and was not able to participate. The 19 participating companies covered large-sized (100**–**200 employees), medium-sized (20–50 employees) and small-sized companies (< 20 employees). An information letter was distributed to all employees and an appointment was made with each company for interviews. The total population consisted of 927 employees. Of practical reasons, all employees present on the day of interview were included. 160 employees were not present the specific day. Of these, 42 were on sick leave and the rest had leave of absence or duty outside the workplace. Workers with poor English or Norwegian language skills were excluded (n = 10). Of the remaining 757 workers, 95% participated in the main study. The subsample used in the current study was male car mechanics (n = 263), a group of workers with relatively homogenous job content. Female car mechanics were excluded because the group was small (n = 4) and the study focused on a working context dominated by men.

Each interview lasted for a maximum of 20 minutes and was carried out in a separate room at the workplace in 2007. IH and one research assistant performed the interviews. IH interviewed and the assistant transcribed during the interviews, using a laptop. The main interviewer (IH) was present at every interview. In addition to information about gender, age, education, number of years in current company and in working life, information about sickness absence was requested. For the present qualitative study the following open-ended question was asked: ‘*What is decisive for choosing absence or attendance when you wake up in the morning, feel ill and wonder whether you should attend work or not?*’ Descriptive statistics regarding demographic data and sickness absence were performed. The open-ended question was analysed with systematic text condensation [20,21]: (i) TM and IH read the transcription of the interviews to form a general view of the themes presented; (ii) units of meaning were identified and coded; (iii) the meaning in the coded groups was condensed, and; (iv) generalized descriptions reflecting the decisive theme for choosing absence or attendance among car mechanics were made.

## Results

Table 1 shows the demographic characteristics of the participants. Half of the participants were between 17 and 30 years old. The mean number of days of self-certified sickness absence the past year was 2.3 (range 0*–*12).

**Table 1:** Description of the study sample (n = 263)

	**Median**	**Mean**	**SD**	**Min.–max.**
Age	29.0	31.8	12.4	17–65
Education (years)	3.6	3.6	1.3	0–7
Years in current company^a^	5.0	8.8	10.0	0–48
Years in working life^b^	8.0	12.2	12.4	0–47

^a^ Apprentice period included.

^b^ Apprentice period not included.

**Figure 1:**
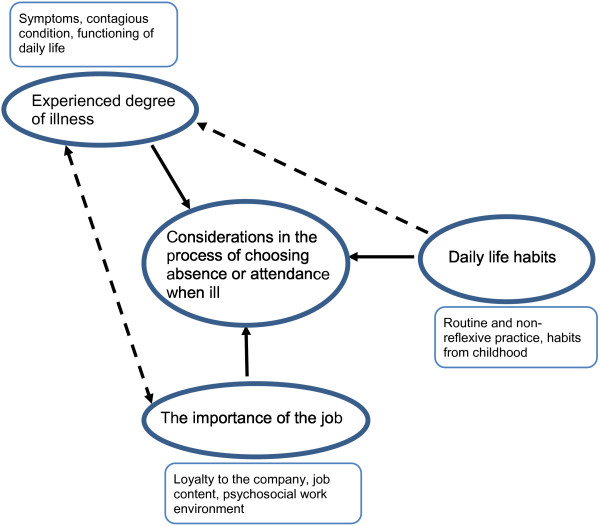
Areas for consideration in the process of choosing absence or attendance when ill.

Among the 263 car mechanics interviewed, eleven persons did not answer on behalf of own experiences, but rather presented general opinions and no information on their own decisions. This was not in accordance with our main research question. For the remaining 252 informants, we found three major themes at stake during the process of choosing absence or attendance when feeling ill (Figure 1);

1) *Experienced degree of illness* – as indicators of whether you are fit for work or not, 2) *daily life habits* – the everyday habit of going to work, no matter what, and 3) *the importance of the job* – the necessity to finish work because of the responsibility towards customers and colleagues. In the following, the three categories with typical quotations will be presented.

### Experienced degree of illness

For many informants, experienced degree of illness was decisive for choosing absence or attendance. The symptoms were interpreted in terms of severity and the severity was given meaning in terms of work ability, consequences for their workplace and problems with functioning in daily life. One participant described his decision related to severity of illness in this way:

To be home, I have to feel really bad, either the back is damaged or I have migraine.’

Some workers worried about having a contagious condition. A car repair process may demand physical closeness of the workers involved, and this work situation was given as an example of possible danger of infecting the colleagues. If they had high fever or an illness that was contagious, then they decided to stay at home. Gastrointestinal complaints like vomiting and diarrhoea were other obvious reasons for staying at home.

The experienced degree of illness was often related to the functioning of daily life, tested for example by the ability to get out of bed, take a shower, dress and eat breakfast. The functional ability to work was evaluated in the perspective of the functional ability to do ordinary things at home. If it was possible to perform daily activities at home, then you could as well go to work. A statement by a man in his fifties illustrates how illness could be linked to functioning:

I stay at home only if I’m not able to get out of bed, for example if my chest is so tight that I have problems breathing – then I cannot work in an upright position.’

A group of workers said that they never reflected upon the question to call in sick, even if they had severe diseases. They perceived themselves as healthy individuals with little experience of illness and no need for calling in sick: ‘I am seldom, almost never, sick, so that thought doesn’t cross my mind.’ One man even told that his health was very good in spite of being diagnosed with rheumatoid arthritis. Even if this disease might frequently involve pain and disability, he never considered this as a reason for staying at home.

### Daily life habits

A number of informants expressed that daily life habits governed the decision of absence versus attendance. The everyday practise of attending work was based on a routine, and not a result of reflection about their well-being. Early morning was not a time for decision-making, more a time of tiredness and unease. The consequences of attending work no matter what, were often reflected upon later. Some informants experienced improved health during the working day while other realised that staying at home would have been better. A typical quote about this behaviour was stated by a man in his thirties:

‘In the morning I do not feel that much, I just go to work. However, arriving at the workplace, I feel I should have stayed at home. I do not think about this in the morning. Then, I am on autopilot.’

Others told us about habits from childhood that they applied in their present work. These informants emphasised that the habits were acquired in the family. One car-mechanic, aged nearly fifty, said:

I always go to work. I’ve been raised believing you should be sick before staying at home. A faint headache or an ordinary cold is no reason for staying at home. From the age of eight I had to work on the farm every morning before school. I got up early every morning to work.’

### The importance of the job

Statements in the third category, the importance of the job, dealt with attendance incentives that pulled the employees towards work. The main incentives were loyalty to the company, job content and psychosocial work environment, with focus on colleagues, customers and company. The statements often addressed the negative consequences of being absent from work. A twenty-year-old man put it this way:

‘… I am thinking…. I’ll have to be at work. There are tasks booked for me that I have to do. If not, we risk losing the customers, and that will affect my colleagues.’

Job satisfaction also promoted work attendance in spite of sickness. Attendance was highly valued and described as a feeling of well-being, making absence less attractive. However, a good psychosocial work environment seemed to be important for this decision. A man who was nearly forty said:

‘I attend work if I have a headache or whatever. I’m satisfied with my work - nice colleagues.’

The opposite view was also expressed, though rarely. A few workers described that job dissatisfaction could bring forth decisions of sickness absence. A thirty-seven-year-old mechanic put it this way:

‘Sometimes I am fed up, there is not much praise here.… That’s one of the reasons for calling in sick, when you’re really not that sick. The job I had before…. was very different, no sickness absence for five years. Job satisfaction is important in relation to absence.’

In general, the informants emphasized either “experienced degree of illness, daily life habits or the importance of the job” when reflecting on their main reasons for choosing absence or attendance when feeling ill. However, during the reflection the two remaining areas were often mentioned as influential. Decisions based on “daily life habits” could be influenced by the “experienced degree of illness”, however, not before the worker had attended work and reflected on his illness. The “experienced degree of illness” was often influenced by “the importance of the job” and brought thus both areas into consideration as part of the decision process.Figure 1 illustrates possible combinations of the three areas of consideration.

## Discussion

This is the first qualitative study that examines decisions of absence versus attendance when feeling ill among male car mechanics. We found three themes at stake during the decision-making process: 1) Experienced degree of illness, 2) daily life habits, and 3) the importance of the job.

The strength of this study is the use of an open-ended question about decisive themes for absence or attendance at work when feeling ill. Such a question can provide a deeper insight into the process of calling in sick than closed answer surveys can. However, because this question was part of a study with several other questions, the possibility of follow-up questions was limited, and the answers may therefore reflect intuitive responses, rather than thorough reflections. The dilemma of choosing absence or attendance when feeling ill may be a relevant issue for other employees. However, the generalizability is limited, as the absence–attendance culture is probably deeply embedded in organisational practice [22]. The study involved only men, and women might have had other experiences and other areas for consideration than what was found among men, for example due to gendered roles in child care and domestic work. In contrast to studies with similar questions, the informants in the current study were not selected because of their illness or sickness absence [18,23]. All informants were present at work and reflected on previous experiences of illness and the possible dilemma of attending work or not. Consequently, we do not know their actions, but rather their attitudes to such situations. Of practical reasons, all workers on sickness absence on the day of interview were not included in the study. This is a limitation, as the current sample has to rely on the memory of reporting ill. The present workers may also be selective for those who decide to attend work even if they feel ill. The workers on sickness absence might differ from the present informants regarding decisions of absence versus attendance and therefore might have offered valuable experiences that would have been particularly relevant for the question in our study. Another possible limitation is related to the finding that informants seldom presented negative sides of themselves, such as socially incorrect behaviour [24]. Hence, there might be a bias due to the informants’ desire to make a good impression.

The first theme identified when considering absence or attendance dealt with the experienced degree of illness as perceived by the informants the morning in question. This is in line with the findings in a qualitative study among offshore catering workers [8], where the participants expressed that the severity of their health conditions, for instance if it was contagious, was decisive for staying away from work. Several informants in our study presented symptoms to illustrate the experienced degree of illness. High fever, vomiting and diarrhoea were considered incompatible with work attendance, thus making a decision of absence versus attendance unnecessary. Decision of work attendance seemed to be based on ability to perform daily life activities at home, which was equated with the ability to work. Therefore, this category may not be specific for car mechanics, but indicate that a norm of legitimate absence is in play. However, the presented illnesses differ from those reported in other studies of presenteeism or absenteeism. The most frequent causes of sickness absence are found to be musculoskeletal pain, fatigue and slight depression [13]. In Norway, causes of absence are dominated by musculoskeletal disorders and mental disorders [25,26]. Among car mechanics, musculoskeletal problems are found to be associated with absence from work [17]. Surprisingly, our informants focused neither on musculoskeletal nor mental disorders. This observation may indicate that the symptoms described are associated with self-certified sickness absence. Although the causes of self-certified sickness absence in Norway are unknown, they are probably dominated by flu and cold [27]. Symptoms of viral infections have often an acute onset and a short duration and seldom require medical treatment. Musculoskeletal and mental disorders more often require medical advice and sickness certification. By consulting a doctor the workers may transfer the decision and potential dilemma of sickness absence versus sickness attendance to a professional.

Daily life habits were the second theme identified in this study. In nearly all cases the outcome of habits was sickness attendance. According to the push-pull model of motivation developed by Gambetta [28], daily life habits may be interpreted as a push factor. Behaviour based on norms, traditions or class values is described as non-reflexive and ‘pushes the individual from behind’. Strict work-attendance standards and internalized work-duty norms seem to promote the sickness attendance practice. This phenomenon is also found in other studies of sickness absence and sickness attendance [24]. Workers with conservative attitudes towards absence have been found to prefer sickness attendance [1]. A study describes the father who ‘never took a day off from work due to illness’ as influential in men’s account of common health problems and work [29]. The typical descriptions of habits in the family and during childhood indicate that the daily life habits may be of general relevance and not related to the specific occupation of car mechanics. However, even though not explicitly expressed by the informants, the individual behaviour is also assumed to be constrained by the social influence and the absence culture of the organization [30].

The third theme, the importance of the job, may be interpreted as a kind of attendance requirement i.e. the work have to be finished if negative consequences for customers and colleagues were to be avoided. According to the push-pull model of Gambetta [28], this fits well into the pull dimension. ‘Pulled from the front’ assumes that individuals act purposely in accordance with their intentions. Faced with multiple options, they will reflect and choose according to anticipated future rewards. In this case career interests and responsibility for work tasks, colleagues and customers may pull the person towards work despite of him feeling ill. Other models use both ‘attendance incentives’ and ‘attendance requirements’ [7] to label mechanisms that resemble the pull and push concepts employed by Gambetta. In the present study we use pull to signify that individuals are pulled towards work because their attendance is highly required [24]. Sickness presenteeism seems to occur in small firms, where time pressure is prevalent and the replacement of an absent employee is difficult. In such an environment sick leave often has a negative consequence for the absentee, his colleagues or third parties [1,6]. The context of car mechanic work strongly reflects these characteristics. The study on offshore catering workers [8] also underscored the importance of specific job situations, for instance if they were onshore or offshore the day of decision. The findings support the general view of the work environment as important for decisions of sickness absence or presence. There is still limited knowledge about the impact of cultures in different occupations, and also possible gender differences. This should be studied further.

Many of the workers that presented themselves as healthy individuals seemed to experience minor illnesses, but did not express a dilemma regarding absence or attendance. It may be that individuals who characterise themselves as healthy hardly ever have health problems, or they may have an unusual high threshold for feeling sick. However, the mean age of 32 years in our study population indicates that these workers represent a healthy group.

The three themes of considerations in the process of choosing absence or attendance when ill are not assumed to be occur separately, even though the informants in our study seemed to have their primary focus on one of the themes. The relationship between “experienced degree of illness” and “the importance of the job” are supposed to be closely related and possibly dependent on the specific job context. Similarly, the “model of illness flexibility” by Johansson & Lundberg [7] describes the role of work specific factors “adjustment latitude” and “attendance requirement” in combination with “loss of function” in the choice between going to work or not when sick. The statements included in “daily life habits” might be expressions of “attendance requirement” that were not reflected on or made explicit by the informants. This hypothesis has to be followed up by more studies.

A strong motivation for work attendance dominated the decision process in all three areas for consideration in our study. The focus on attendance versus absence in case of sickness may be influenced by low age and a ‘healthy worker effect’ i.e. the phenomenon that unhealthy individuals are excluded from demanding work, whereas healthy individuals remain [31]. However, the sickness absence among car mechanics is about the same as the mean sickness absence among Norwegian men [27], which does not support the healthy worker effect.

## Conclusions

The car mechanics in the current study expressed a strong will to attend work and was reluctant to make the decision of calling in sick for work. We revealed three main themes at stake during the decision-making process of choosing absence or attendance when feeling ill: 1) Experienced degree of illness, 2) daily life habits, and 3) the importance of the job. Knowledge of attitudes and dilemmas in decisions regarding sickness absence and attendance, as added in this study, is crucial for promoting work attendance, avoiding harmful sickness attendance as well as harmful sickness absence. These decisions should be investigated further in other occupational contexts and among women.

## Competing interests

The authors declare that they have no competing interests.

## Authors’ contributions

IH conceived the study and performed the interviews. All authors analysed the data, drafted and edited drafts of the manuscript. All authors read and approved the final manuscript.

## Pre-publication history

The pre-publication history for this paper can be accessed here:

http://www.biomedcentral.com/1471-2458/12/813/prepub
